# Evaluation of the Mucosal Immunity Effect of Bovine Viral Diarrhea Virus Subunit Vaccine E2Fc and E2Ft

**DOI:** 10.3390/ijms24044172

**Published:** 2023-02-20

**Authors:** Yanqing Cheng, Shaoyu Tu, Tong Chen, Jiahui Zou, Sheng Wang, Meijun Jiang, Shan Tian, Qingli Guo, Sizhu Suolang, Hongbo Zhou

**Affiliations:** 1 State Key Laboratory of Agricultural Microbiology, College of Veterinary Medicine, Huazhong Agricultural University, Wuhan 430070, China; 2 Key Laboratory of Preventive Veterinary Medicine in Hubei Province, The Cooperative Innovation Center for Sustainable Pig Production, Wuhan 430030, China; 3 Department of Animal Science, Tibet Agricultural and Animal Husbandry College, Nyingchi 860000, China

**Keywords:** BVDV, subunit vaccine, mucosal vaccination, molecular adjuvants, Fc receptor, ferritin

## Abstract

Classified as a class B infectious disease by the World Organization for Animal Health (OIE), bovine viral diarrhea/mucosal disease is an acute, highly contagious disease caused by the bovine viral diarrhea virus (BVDV). Sporadic endemics of BVDV often lead to huge economic losses to the dairy and beef industries. To shed light on the prevention and control of BVDV, we developed two novel subunit vaccines by expressing bovine viral diarrhea virus E2 fusion recombinant proteins (E2Fc and E2Ft) through suspended HEK293 cells. We also evaluated the immune effects of the vaccines. The results showed that both subunit vaccines induced an intense mucosal immune response in calves. Mechanistically, E2Fc bonded to the Fc γ receptor (FcγRI) on antigen-presenting cells (APCs) and promoted IgA secretion, leading to a stronger T-cell immune response (Th1 type). The neutralizing antibody titer stimulated by the mucosal-immunized E2Fc subunit vaccine reached 1:64, which was higher than that of the E2Ft subunit vaccine and that of the intramuscular inactivated vaccine. The two novel subunit vaccines for mucosal immunity developed in this study, E2Fc and E2Ft, can be further used as new strategies to control BVDV by enhancing cellular and humoral immunity.

## 1. Introduction

The bovine viral diarrhea virus (BVDV), together with the border disease virus (BDV) and swine fever virus (CSFV), belongs to the *Pestivirus* genus of the Flaviviridae family [1]. A combination of persistent infection (PI) and cytopathic infection in calves often results in severe mucosal disease, with mortality up to 100% [2]. BVDV distributes widely in the world, with a relatively high antibody-positive rate. In China, prevalent types are 1B, 1M, and 1Q of the type-1 BVDV [3,4,5]. Phylogenetic analysis revealed that bovine viral diarrhea virus type 1 was imported into China in the 1960s and that at least eight BVDV-1 genotypes are in circulation in China. Among them, 1B and 1m are the major genotypes [6]. This is consistent with our epidemiological investigation of BVDV in Tibet.

The spherical virion of BVDV is approximately 40–60 nm in diameter and is enveloped by a capsule. The single plus-stranded viral RNA genome of BVDV is approximately 12.5 KB in length. The viral polyprotein is subsequently cleaved by a combination of host and viral proteases into four structural (C, E0, E1, E2) and eight nonstructural (Npro, P7, NS2, NS3, NS4A, NS4B, NS5A, NS5B) proteins [7,8]. BVDV-E2 possesses strong immunogenicity, and evidence has shown that neutralizing antibodies (NAbs) induced in infected animals mainly target E2 [9]. Meanwhile, multivalent E2 subunit vaccines targeting major circulating strains of BVDV can provide partial protection against homologous strains. A recombinant herpesvirus vaccine constructed with BVDV E2 protein and bovine herpesvirus 1 (BoHV-1) D protein induced specific immune responses to both viruses in the host after intranasal nebulization [10]. Therefore, it might be a good strategy to control BVDV by employing the immunodominant BVDV E2 protein as a subunit vaccine.

The nasal cavity is rich in lymphocytes and antigen-presenting cells, provides a suitable microenvironment for mucosal immunization, and protects protein vaccines from being destabilized by acids and alkalis. In addition, intranasal vaccine-induced immunoglobulin A (IgA) and resident memory B and T lymphocytes in the upper respiratory tract are capable of effectively blocking viral replication [11]. Immunization through the nasal cavity not only induces mucosal immunity but also stimulates systematic immune responses, which exert immune protective effects. Previous studies have found that antigens could be completely delivered into the mucosal epithelium by targeting subunit vaccines to the neonatal Fc receptor (FcRn) of M cells on the mucosal epithelium, thereby effectively stimulating specific immune responses [12,13,14]. The immunization effects of nasal spray are significantly enhanced by utilizing a soluble recombinant vaccine formed by the fusion of molecular adjuvant and immune antigen [15]. IgGFc interacts with FcRn on the surface of immune cells to participate in the transport of IgG through mucosal surfaces, improving the immunogenicity of Fc-fused proteins in mammals [16]. IgGFc fusion proteins were reported to form stable dimers through disulfide bonds in the Fc hinge region to increase the half-life and stability of recombinant proteins, therefore improving the cellular, mucosal, and humoral immune responses [17,18]. Novel vaccines mediated by the IgGFc have been successfully applied to the influenza A virus (HA-HuFc) [19] and swine fever virus (CSFV-E2Fc) [20].

The appropriate surface modification enables nanoparticles to reside stably in the blood circulatory system [21]. Characterized by a large surface area and a hollow spherical structure, Ferritin (Ft) was reported to form nanoparticles of 24 identical polypeptides by self-assembling with a variety of expressed fusion proteins, which promoted antigen recognition and uptake by antigen-presenting cells, thereby inducing a strong immune response [22,23]. Ferritin is widely applied in vaccine development. Covalent integration of the receptor-binding domain (RBD) antigen of the SARS-CoV-2 spike protein and self-assembled Helicobacter pylori non-heme ferritin results in a potent production of neutralizing antibodies. Ferritin protein nanoparticles also have a strong ability to improve antigen stability, overcome biological barriers, and achieve targeted delivery. As nano-vaccines can be efficiently captured by dendritic cells and macrophages [24], ferritin nanoparticles are a new and promising platform for antigen delivery.

Currently, the main available vaccines for BVDV are inactivated vaccines and subunit vaccines, both of which require time-consuming and labor-intensive vaccination by intramuscular injection. Meanwhile, inactivated vaccines produced through MDBK cells are prone to induce bovine neonatal pancytopenia (BNP) [25]. However, subunit vaccines have shown superior safety profiles [26]. Intranasal immunization has been shown to target antigens to the FcγR receptor, causing mucosal and systemic immune responses [15,16]. However, the immune effects of intranasal immunization with BVDV vaccines and the effectiveness of BVDV-E2 combined with molecular adjuvants (IgGFc and Ft) remain poorly studied. Therefore, the development of mucosal vaccines with BVDV-E2 protein to improve their immune efficacy is a top priority in this field. In the current study, E2 protein-fused Fc or Ft subunit vaccines were generated in HEK293 cells. The findings showed that nasal vaccination with the BVDV E2Fc subunit vaccine was more effective than nasal vaccination with the E2Ft subunit vaccine, further supporting the indications that the E2Fc fusion protein is a more potent subunit vaccine option.

## 2. Results

### 2.1. The Expression of Recombinant Proteins in Suspended HEK293 Cells

**Figure 1:**
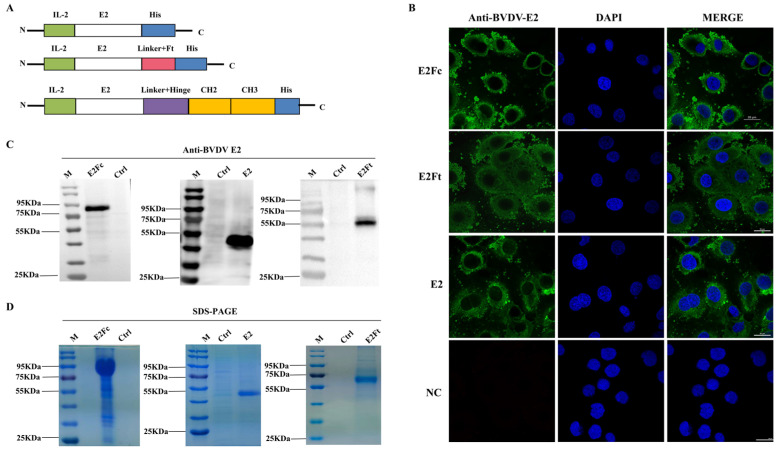
Immuno-expression and identification of E2, E2Ft, and E2Fc proteins in HEK293 cells. (**A**) The construction diagrams of BVDV-E2, BVDV-E2Ft, and BVDV-E2Fc. (**B**) Detection of the immuno-expression of recombinant proteins by IFA; 48 h after transfected with E2, E2Fc, or E2Ft, HEK293 cells were fixed for IFA. BVDV E2 monoclonal antibody and FITC-goat anti-mouse IgG were applied as primary and secondary antibody, respectively. Nucleus was stained with DAPI (blue). Scale bar represents 20 μm. (**C**) Detection of purified recombinant proteins by Western blot. (**D**) Identification of purified recombinant proteins by SDS-PAGE.

Structural diagrams of E2, E2Ft, and E2Fc are shown in Figure 1A. Indirect immunofluorescence assays were first conducted using preserved monoclonal antibody (mAb) BVDV-E2 to verify the immuno-expression of recombinant fusion proteins. Cells transfected with plasmids containing E2, E2Ft, or E2Fc genes emitted a fluorescent signal, while mock-treated cells did not exhibit a fluorescent signal (Figure 1B). This indicated strong expression of E2, E2Ft, and E2Fc in HEK293 cells. To determine whether these proteins were secretory expressed, the supernatants of HEK293 cells transfected with plasmids containing E2, E2Ft, or E2Fc genes were collected after 6 days. The immuno-expression levels of each protein were analyzed by Western blot with BVDV-E2 antibody. The results demonstrated that cells transfected with plasmids containing genes of E2, E2Ft, and E2Fc expressed corresponding proteins with molecular masses of 45 kDa, 60 kDa, and 80 kDa, respectively (Figure 1C). SDS-PAGE and Coomassie blue staining results showed that the bands of each sample were consistent with the blotting results (Figure 1D). In order to ensure the specificity and accuracy for detecting the protein immuno-expression, pre-absorption assays of BVDV-E2 mAb were first conducted and followed by viral replication assays and Western blot to testify the specificity of BVDV E2 mAb. The results revealed that the antibody can only react to BVDV-E2 protein (Figure S1). Taken together, the evidence showed that recombinant proteins E2, E2Fc, and E2Ft were expressed abundantly in HEK293 cells.

### 2.2. Activation of the APC-FcγRI by BVDV-E2Fc

**Figure 2:**
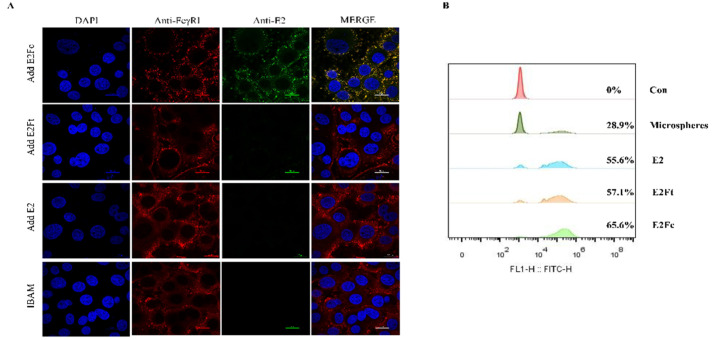
Activation of the APC-FcγRI by BVDV-E2Fc. (**A**) Co-localization analysis of CD64 (FcγRI) with E2Fc protein on BAM was detected by confocal microscopy. CD64 (FcγRI) on BAM was stained with rabbit anti-CD64 and Cy3-labeled goat anti-rabbit IgG as the primary and secondary antibody, respectively. E2Fc was stained with mouse anti-E2 monoclonal antibody and Alexa Fluor^®^ 488 goat anti-mouse IgG as the primary and secondary antibody, respectively. Nucleus was stained with DAPI (blue). Images were merged to analysis the co-localization (yellow) of CD64 (FcγRI) with E2Fc. Scale bar represents 20 μm. (**B**) Flow cytometry analysis of phagocytosis rate of E2 proteins on BAM. BAM were pre-incubated with E2, E2Ft, or E2Fc protein for 4 h. Cells were incubated with 10 μL FITC-conjugated microspheres for 2 h. Adherent macrophages were digested with trypsin and resuspended with PBS. Fluorescence intensity at 488 nm was determined by the Beckman flow cytometer.

Antigen recognition of BVDV-E2Fc (E2, E2Ft) by macrophages plays an important role in the activation of the immune response. In order to detect the activation effect of the fusion protein on the immune system, the recognition function and phagocytic activity of the fusion protein by bovine alveolar macrophages (BAM) were verified in vitro. The results showed that CD64 (FcγRI), detected by Cy3-labeled goat anti-rabbit IgG antibody (red) markers, was localized in the cytoplasm and cell membrane in bovine macrophages. E2Fc was detected using goat anti-mouse IgG (green fluorescence). Images were merged for the analysis of the co-localization of CD64 (FcγRI) and E2Fc in bovine alveolar macrophages (yellow color; Figure 2A). In contrast, CD64 did not co-localize with E2 or E2Ft. The results showed that translocation of the E2Fc fusion protein across the mucosal barrier was mediated by the FcγRI.

Phagocytosis, the uptake of particles by macrophages, was measured as the phagocytosis rate. The phagocytosis rates of FITC-conjugated microspheres, E2 protein, E2Ft, and E2Fc by the bovine alveolar macrophages were 28.9%, 55.6%, 57.1%, and 65.6%, respectively. In other words, the phagocytosis rate of E2Fc was higher than that of E2Ft, which further proved that the capture of antigens by monocytes was completed by the binding of a specific receptor on its surface with corresponding ligands. However, bovine alveolar macrophages possessed no receptors for E2 and E2Ft proteins, and they only removed foreign bodies through phagocytosis, which is the most basic defense method of the body (Figure 2B).

### 2.3. Potent Mucosal and Humoral Immune Responses Induced by E2Fc In Vivo

**Figure 3:**
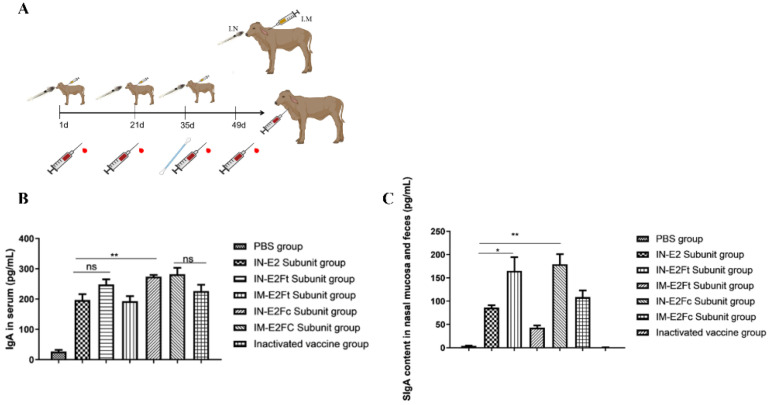
The calf immunization experiments flow chart and mucosal and humoral immune responses induced by E2Fc in vivo. (**A**) BVDV subunit vaccine construction and calves immunization experiment flow chart. (**B**,**C**) The measurement of (**B**) IgA and (**C**) secretory IgA levels in the nasal swabs, feces, and serum samples collected on day 14 after secondary immunization by ELISA. *n* = 5, * *p* < 0.05, and ** *p* < 0.01.

The calf immunization procedure is depicted in Figure 3A. The levels of IgA and SIgA produced by calves immunized with the fusion protein were detected by indirect ELISA. Serum, feces, and nasal mucosa samples were collected and detected by a commercial ELISA kit. The E2Fc-immunized group exhibited the highest levels of IgA and SIgA in serum and mucosa, both in intramuscularly and mucosal-immunized animals (*p* < 0.01) (Figure 3B,C). No significant differences in IgA levels were observed between the E2-immunized group and the E2Ft-immunized group, but they did differ in the SIgA levels (*p* < 0.05). In conclusion, the E2Fc fusion protein was able to induce more potent mucosal and humoral immune responses than other proteins.

### 2.4. E2Fc Can Induce Stronger T-Cell Immune Responses (Th1 Type) against BVDV Compared with E2Ft In Vivo

**Figure 4:**
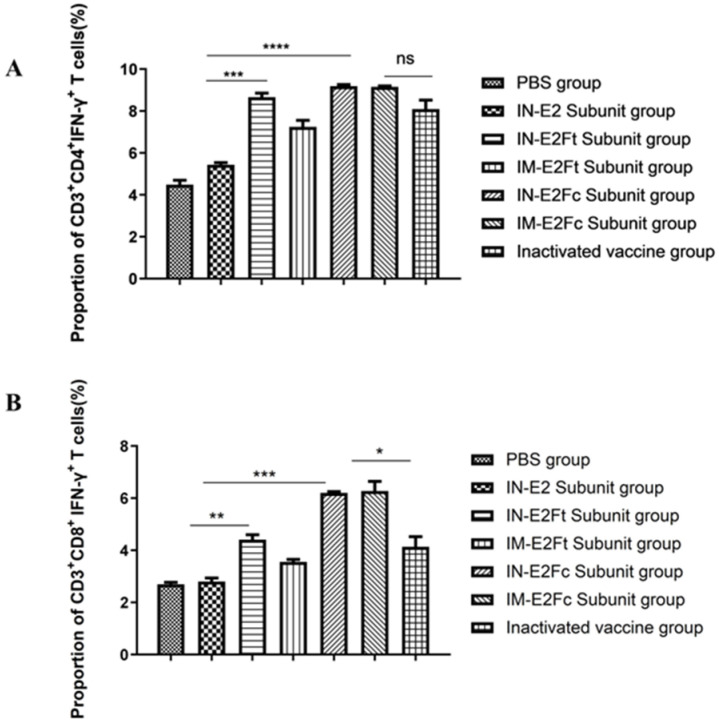
T-cell immune responses against the BVDV in vivo. Percentage of (**A**) CD4^+^ IFN-γ+ T cells and (**B**) CD8^+^ IFN-γ+ T cells after stimulation in bovine peripheral blood mononuclear cells. *n* = 5, * *p* < 0.05, ** *p* < 0.01, *** *p* < 0.001, and **** *p* < 0.0001.

The Th1-type cytokine IFN-γ expressed by T cells and NK cells mediate cellular immunity, which is essential for virus clearance. To detect the distribution of IFN-expressing CD4^+^ and CD8^+^ T cells in calves, lymphocytes were isolated from the PBMC of calves 35 days post immunization. Lymphocytes were stimulated with inactivated BVDV, and flow cytometry was subsequently applied to identify the IFN-γ-secreting CD4^+^ and CD8^+^ T cells. Mucosa-immunized groups with E2Fc and E2Ft exhibited significantly higher proportions of IFN-γ-secreting CD4^+^ and CD8^+^ T cells compared with the E2 group. The group that was mucosa-immunized with E2Fc had a higher level of IFN-γ-secreting CD3^+^ derived CD8^+^ T cells than the group immunized with inactivated BVDV (*p* < 0.05) (Figure 4). However, no significant difference in IFN-γ-secreting CD3^+^ derived CD4^+^ T cells was observed between the groups with mucosa-immunized E2Fc and with inactivated BVDV. In summary, both intramuscular injection and mucosal immunization with E2Fc induce stronger T-cell immune responses (Th1 type) against the BVDV compared with E2Ft.

### 2.5. Immunization with Recombinant Subunit Vaccines Increases the Content of Th1-Type Cytokines

**Figure 5:**
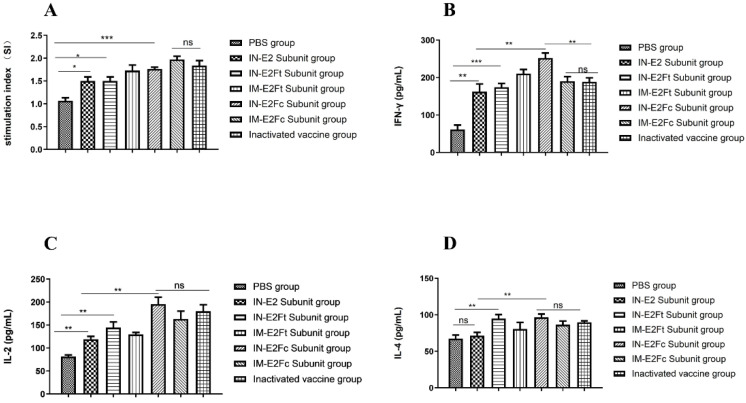
The content increase of Th1-type cytokines after immunization with recombinant subunit vaccines. (**A**) The cell proliferation of peripheral blood lymphocytes of calves after immunization 49 days was detected by lymphocyte proliferation assay kit. (**B**–**D**) Th1 and Th2 cytokines in lymphocyte supernatants were detected by bovine IFN-γ, IL-2, IL-4 double-antibody sandwich ELISA Kit. *n* = 5, * *p* < 0.05, ** *p* < 0.01, and *** *p* < 0.001.

To verify the effect of recombinant subunit vaccine on cellular immune responses, we analyzed lymphocyte and cytokine proliferation levels in the peripheral blood of immunized calves. First, anticoagulants were collected from calves 49 days after the immunization. Lymphocytes were stimulated by the ultraviolet-inactivated BVDV after separation, and correlated indices were measured. Compared with the PBS group, the lymphocyte stimulation indices (SI) of groups immunized with the subunit vaccines (E2, E2Fc, and E2Ft) were all increased to different degrees, with the highest lymphocyte stimulation index (*p* < 0.001) in the E2Fc-immunized and the inactivated vaccine groups (Figure 5A). In addition, commercial double antibody sandwich ELISA kits against bovine IFN-γ, IL-2, and IL-4 were applied to detect Th1- and Th2-type cytokine levels in the peripheral blood of immunized calves. Compared with PBS-treated calves, the levels of IFN-γ and IL-2 expressed in immunized calves were significantly higher than IL-4 at 49 days post-immunization. The levels of IFN-γ were higher in the IN-E2Fc group than in the inactivated vaccine group (*p* < 0.05), but there was no difference in IL-2 and IL-4 between the IM-E2Fc and the inactivated vaccine groups (Figure 5B–D). These results suggested that the IN-E2Fc subunit vaccine significantly promoted the Th1-type cellular immune response in calves.

### 2.6. BVDV-Specific Antibodies Can Be Strongly Induced by Recombinant E2Ft and E2Fc Subunit Vaccines In Vivo

**Figure 6:**
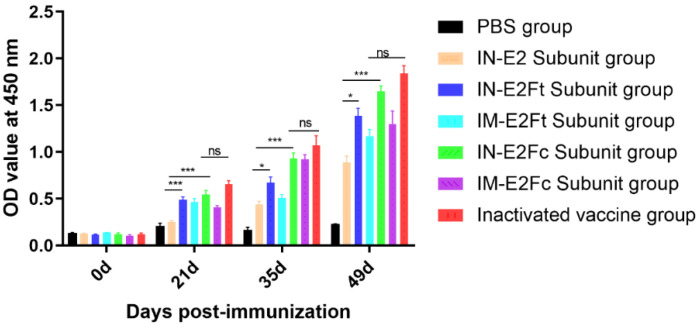
Detection of antibodies in the serum samples of immunized calves by ELISA at day 0, 21, 35, and 49 after immunization. *n* = 5, * *p* < 0.05 and *** *p* < 0.001.

BVDV-specific antibodies were detected by ELISA in order to explore the humoral immune response induced by the E2, E2Ft, and E2Fc subunit vaccines. BVDV-specific antibodies could not be detected in all calves before immunization. The levels of antibodies induced by E2Ft and E2Fc subunit vaccines were significantly higher than those of the E2 group 21 days after the initiation (first dose) of immunization (*p* < 0.001). Antibody levels for mucosal immunity at 35 days (14 days after the second dose) and 49 days (14 days after the third dose) differed significantly between the E2Ft and E2 groups (*p* < 0.05). In addition, E2Fc was significantly higher than E2 (*p* < 0.001). The antibody level of the mucosal immunization group (IN-E2Ft and IN-E2Fc groups) was higher than that of the intramuscular injection group (IM-E2Ft and IM-E2Fc groups), and the antibody level of the inactivated vaccine group was not significantly different from that of the IN-E2Fc group (Figure 6). Taken together, recombinant E2Ft and E2Fc subunit vaccines were able to induce strong humoral immune responses, and mucosal immunity was superior to that of the intramuscular injection, indicating that recombinant E2Fc and E2Ft subunit vaccines were highly antigenic.

### 2.7. Neutralizing Antibodies Against BVDV Induced by Recombinant Subunit Vaccines In Vivo

**Figure 7:**
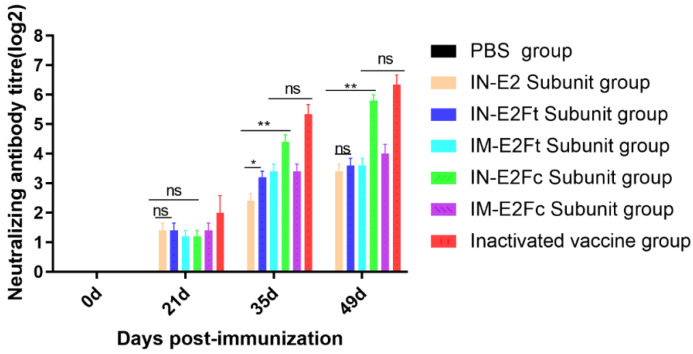
Detection of neutralizing antibodies in the serum samples of immunized calves at day 0, 21, 35, and 49 after immunization. Inactivated serum samples were ratio diluted and pre-incubated with 200 TCID_50_ BVDV XZ02 for 1 h. Serum samples were subsequently added to cells in 96-well plates and incubated for 72 h. Rabbit anti-E2 antibody and FITC-conjugated goat anti-rabbit IgG were employed as the primary and secondary antibody. *n* = 5, * *p* < 0.05, and ** *p* < 0.01.

Blood samples were collected from the jugular vein of calves at 0, 21, 35, and 49 days after the first immunization. Serum samples were separated aseptically and inactivated in a water bath at 56 °C for 30 min. Neutralization assays were conducted to determine the antibody levels in the separated serum. The neutralizing antibodies against BVDV were detected in mucosal-immunized groups with recombinant subunit vaccines, with the titer of the E2Fc neutralizing antibody up to 1:64. This is comparable with the inactivated vaccine and better than the intramuscularly injected E2Ft and E2Fc recombinant subunit vaccines (Figure 7).

## 3. Discussion

It has been reported that the E2 protein expressed in mammalian cells is more effective in the neutralizing protection against BVDV than the E2 protein obtained from the baculovirus insect expression system (brE2) [27]. The suspension culture of mammalian cells is characterized by a simpler process and a higher yield [28]. Suspended HEK293 cells are capable of growing under serum-free conditions, and the cell number is multiple times greater than that of the adherent culture, which can be used for the large-scale production of antibodies [29]. The expression efficiency of pcDNA3.1 combined with the eukaryotic suspension cell transient expression system applied in this study was relatively high, and the recombinant protein could be collected six days post-transfection, with a concentration up to 30 mg·L^−1^, which was sufficient for subsequent animal application.

The bovine viral diarrhea virus mainly invades the upper respiratory tract and the digestive tract, which causes rhinorrhea, coughing, shortness of breath, body temperature elevation, and leukopenia, and it is followed by viremia once the virus enters the blood. Therefore, inhaled intranasal vaccination could provide more effective protection than vaccines that are directly delivered to virus infection sites. Recently, it was shown that intranasal immunization can present antigens to FcγR receptors and elicit mucosal and systematic immune responses [16]. Furthermore, bovine IgG1Fc was capable of binding to the bovine FcγR, a high-affinity receptor for IgG [30]. Our results provided evidence that the Fc from bovine IgG1 could bind with FcγRI on bovine alveolar macrophages (BAM) to achieve transmucosal transport. This could prolong the longevity of the fusion protein in the body and promote the production of specific antibodies. Our results revealed that the level of SIgA induced by mucosal-immunized E2Fc was significantly higher than that of the mucosal-immunized E2 (*p* < 0.01). However, only a small difference was observed between the intramuscular-injected E2Fc group and the mucosal-immunized E2 group. This may be caused by the fact that the small intestine mucosa and the mesenteric lymph nodes would both release a tiny amount of SIgA in response to intramuscular injection with E2Fc adjuvanted with 1313VG. The levels of IFN-γ elicited by mucosa-immunization with E2Fc were significantly higher than those of mucosal immunization with E2 (*p* < 0.01), intramuscular immunization with E2Fc, and mucosal immunization with E2Ft. The levels of IL-2 elicited by mucosa-immunization with E2Fc were significantly higher than those of mucosal immunization with E2, and intramuscular immunization with E2Ft (*p* < 0.01). In the next step, the overall antibody levels were detected after the third immunization. The results showed that the mucosal immunization with E2Fc induced significantly higher levels of overall antibodies compared with the mucosal immunization with E2 (*p* < 0.001), whereas the antibody levels induced by the intramuscularly injected E2Fc and mucosal-immunized E2 were comparable to the intramuscularly injected E2Ft group. The overall immune effects of the mucosal immunization of the E2Fc subunit vaccine were superior to those of the intramuscular immunization. Mucosal immunization of E2Fc not only induced the secretion of SIgA but also elicited specific Th1-type cellular immune response and humoral immune response.

It was reported that the CSFV E2 glycoprotein displayed on the surface of ferritin nanocages induced potent expression of the innate immune cytokines IL-4 and IFN-γ in rabbits [31]. A nanoparticle vaccine developed by covalently binding self-assembled ferritin to the receptor binding domain (RBD) of SARS-CoV-2 was also proved to elicit a robust humoral and cellular immune response [24]. The nasal cavity is rich in mucosa-associated lymphoid tissue, and we showed that the SIgA levels induced by mucosal-immunized E2Ft were higher than those of E2 (*p* < 0.05). CD4^+^ and CD8^+^ T cells were significantly higher in the mucosal-immunized E2Ft group than in the E2 group (*p* < 0.01). CD4^+^ and CD8^+^ T cells play a vital role in the defense against viral infection [32]. Neutralization test and antibody assay results also showed that mucosal immunization with E2Ft was more effective than immunization with intramuscular E2Ft and E2. This was probably because the antigen recognition and presentation system of the organism effectively phagocytosed ferritin (E2Ft) with 24 subunit polymers, resulting in a sensitive and effective antibody response in vivo.

Antibodies are secreted by plasma cells, and neutralizing antibody levels are an important indicator for the detection of immune effects. When the neutralizing antibody potency of the BVDV vaccine is greater than 1:24, it indicates that the vaccine is immunogenic [33]. In this study, neutralization assays were performed to evaluate the neutralizing antibody levels induced by E2 recombinant proteins. Therefore, the jugular venous blood of immunized calves was collected to detect the level of IgG antibodies in serum. The results showed that all immunized calves produced different degrees of neutralizing antibodies at 14 days post the second immunization. The neutralizing antibody levels in calves of the mucosal-immunized E2Fc group and the inactivated vaccine group increased significantly and remained at 1:64 at 14 days after the third immunization (neutralizing antibody titers were detected until the sixth week). However, it is worth noting that the neutralizing antibody levels of mucosal-immunized E2 and E2Ft and intramuscular-immunized E2Fc and E2Ft groups were maintained between 1:8 and 1:16. We infer that antigens of the E2Fc recombinant vaccine are released slowly by the Fc receptor, therefore causing prolonged humoral and cellular immune responses. In contrast, the other four subunit vaccines reside shortly in the body due to the lack of a corresponding receptor. Hence, a booster vaccination or a change of adjuvant may be needed to achieve the expected immune effects.

## 4. Materials and Methods

### 4.1. Virus and Cells

Type 1b BVDV XZ02 strain (GenBank accession number: MF278652) was isolated from Tibet and cultured using Madin-Darby bovine kidney cells (MDBK). Porcine epidemic diarrhea virus (PEDV) was preserved in our laboratory. MDBK cells were purchased from ATCC (ATCC- CCL-22) and maintained in the DMEM (Cat NO. SH302403.01, HyClone, Shanghai, China), and bovine alveolar macrophage cells (BAM) were maintained in the RPMI-1640 (Cat NO. SH30027, Cytiva, Shanghai, China); all the mediums contained 10% heat-inactivated horse serum (Cat NO. 16050122, Gibco, Carlsbad, CA, USA,) and were incubated at 37 °C in a humidified incubator with 5% CO_2_. Suspended HEK293 cells were purchased from ATCC (ATCC CRL-1573) and cultured in a serum-free KOP293 medium (Cat NO. M21201121A, KAIRUI -biotech, Zhuhai, China).

### 4.2. Antibodies and Test Kits

The following antibodies and test kits were used in this study: anti-BVDV E2 antibody (Cat NO. orb312169, Biorbyt, Cambridge, UK); HRP-goat anti-mouse antibody (Cat NO. ab205719, Abcam, Cambridge, UK); Cy3-goat anti-mouse antibody (Cat NO. AS008, ABclonal, Boston, MA, USA); FITC-goat anti-mouse antibody (Cat NO. ab150117, Abcam, Cambridge, UK); CD64 (FcγRI) antibody (Cat NO. abs135850, Absin, Shanghai, China); Alexa Fluor^®^ 488 goat anti-mouse IgG antibody (Cat NO. ab150113, Abcam, Cambridge, UK); Cy3-goat anti-rabbit IgG antibody (Cat NO. ab6939, Abcam, Cambridge, UK); FITC-conjugated microspheres (Cat NO. 17155, Polysciences, Warrington, PA, USA); FITC-CD4 antibody (Cat NO. MCA1653, BIO-RAD, Hercules, CA, USA); PE-CD8 antibody (Cat NO. MCA837, BIO-RAD, Hercules, CA, USA); IFN-γ antibody (Cat NO. MCA1783A, BIO-RAD, Hercules, CA, USA); FITC-goat anti- rabbit IgG antibody (Cat NO. AS011, ABclonal, Boston, MA, USA). Kits and Reagents include: BVDV-E2 antibody detection Kit (Cat NO. SU-AN78401, Win-win Biotechnology Co., LTD, Shanghai, China); IgA detection Kit (Cat NO. 177090b, CAMILO, Nanjing, China); SIgA detection Kit (Cat NO.177053b, CAMILO, Nanjing, China); IFN-γ detection Kit (Cat NO. 177020b, CAMILO, Nanjing, China); IL-2 detection Kit (Cat NO. 177028b, CAMILO, Nanjing, China); IL-4 detection Kit (Cat NO. 1770131b, CAMILO, Nanjing, China); TA-293 transfection reagent (Cat NO. R21203107A, KAIRUI biotech, Zhuhai, China); Lymphocyte separation medium (Cat NO. RNBJ9540, SIGMA, Saint Louis, MO, USA).

### 4.3. Plasmids Construction

Truncated E2 (denoted as E2 hereafter) was generated by removing the transmembrane region of E2 (gene sequence accession number: MF278652) using the TMHMM web tool (http://www.cbs.dtu.dk/services/TMHMM/ accessed on 12 January 2023) and adding a histidine tag to the C-terminus (Figure 1A). The gene sequence is shown in Supplementary Material S1. E2Fc plasmid was constructed by adding an Fc fragment containing the hinge region of the bovine IgG1 (accession number: x62916.1) to the C-terminal of the E2 with the ordinal position of E2-synthetic flexible peptide-hinge region-CH2-CH3 domain-6 × His [20]. The gene sequence is shown in Supplementary Material S2. The E2Ft plasmid was constructed by linking E2 to the Helicobacter pylori ferritin (residues 5–167) via the linker (Gly)_3_Ser [34]. The gene sequence is shown in Supplementary Material S3. The IL-2 signal peptide was added to the N-terminal of E2, E2Fc, and E2Ft to facilitate secretory expression [35]. The target genes (E2, E2Ft, and E2Fc) were cloned into pcDNA3.1 with *Bam*HⅠ and *Xho*Ⅰ, respectively.

### 4.4. Protein Expression and Purification

Approximately 100 mL of suspended HEK293 cells at the exponential stage (approximately 2~4 × 10^6^ cells/mL) were transfected with corresponding plasmids. Briefly, 5ml KPM (transfection buffer) and 100 μg of sterile plasmid DNA were added to a sterile centrifuge tube, and 5 mL of KPM and 500 μL of TA-293 (Cat NO. R21203107A, KAIRUI biotech, Zhuhai, China) transfection reagents were added to another sterile centrifuge tube. Then, a diluted transfection reagent was added to the diluted plasmid and mixed thoroughly. After incubation for 10 min at room temperature, a DNA-transfection reagent complex was added to cells, accompanied by shaking. Cells were incubated in a humidified incubator at 37 °C with 5% CO_2_ at a shaking speed of 140 rpm/min. Cells were harvested six days post-transfection by centrifugation for 30 min at 12,000 rpm/min and filtered with a 0.22 mm filter to remove residual cell debris. The nickel affinity chromatography columns (Cat NO. 17531801, GE, Boston, MA, USA) were employed for histidine (His) tagged affinity purification of E2, E2Ft, and E2Fc proteins [36]. Briefly, 100 mL samples were added to affinity columns at a rate of 1 mL/min. The columns were subsequently washed with five column volumes (CV) of buffer A (0.1 M PBS, 300 mM NaCl, 5 mM) at a rate of 1 mL/min. Finally, concentration gradients of imidazole (from 0 to 500 mM) were allowed to flow through the column at a rate of 1 mL/min to collect the liquid corresponding to the main peak. Protein concentrations were quantified with a BCA protein detection kit. Collected proteins were further used for Western blot analysis.

### 4.5. Western Blot

Proteins were separated using SDS-PAGE. After being transferred to the NC filter membrane, proteins were blocked with 2% skimmed milk powder overnight at 4 °C and subsequently incubated with anti-BVDV E2 antibody (Cat NO. orb312169, Biorbyt, Cambridge, UK) for 2 h at room temperature. After being washed three times with TBS-T, the membranes were incubated with HRP-goat anti-mouse (Cat NO. ab205719, Abcam, Cambridge, UK) antibodies for 1 h at room temperature. After being washed three times with TBS-T, the ECL chemiluminescence system was employed to detect protein immuno-expression.

### 4.6. Indirect Immunofluorescence

For immunofluorescence detection of the immuno-expression of recombinant proteins, HEK293 cells were transfected with plasmids expressing E2, E2Ft, and E2Fc; 48 h after transfection, cells were fixed with paraformaldehyde for 15 min. After being washed three times with PBS, cells were permeabilized with 0.5% Trition-X100 for 15 min. After an intensive wash with PBS, cells were subsequently blocked with 2% BSA and incubated with a 1:500 diluted BVDV E2 monoclonal antibody (prepared in our laboratory) at 37 °C for 2 h. Cy3 (Cat NO. AS008, ABclonal, Boston, MA, USA) or FITC-conjugated goat anti-mouse antibodies (Cat NO. ab150117, Abcam, Cambridge, UK) were applied as the secondary antibody. The nuclei were stained with DAPI for 10 min at room temperature. Proteins were visualized by confocal microscopy (LSM 510, Zeiss, Oberkochen, Germany) [37].

### 4.7. APC-Targeting of the E2Fc Recombinant Fusion Protein

Immunofluorescence colocalization analysis was performed to confirm the binding of the BVDV-E2Fc fusion protein to the FcγRI on bovine alveolar macrophages (BAM). Briefly, 3 µg of purified E2, E2Ft, or E2Fc were added to bovine alveolar macrophages. After incubation for 12 h at 37 °C, cells were washed with 0.1% BSA in phosphate-buffered saline (PBS) and fixed with methanol/acetone (1:1) at 20 °C. Cells were subsequently permeabilized with 0.5%Trition-X100 for 15 min and blocked with 2% BSA at 37 °C for 2 h. Then, the cells were washed three times with PBS and incubated with the BVDV E2 monoclonal antibody (mouse source; prepared in our laboratory) and bovine CD64 (FcγRI) polyclonal antibody (Cat NO. abs135850, Absin, Shanghai, China) for 2 h. The cells were washed three times with PBS and covered with Alexa Fluor^®^ 488 goat anti-mouse IgG (Cat NO. ab150113, Abcam, Cambridge, UK) and Cy3-labeled goat anti-rabbit IgG antibody (Cat NO. ab6939, Abcam, Cambridge, UK) for 1 h at room temperature. The nuclei were stained with DAPI. The images were photographed using immunofluorescence colocalization (LSM 880, Zeiss, Oberkochen, Germany).

### 4.8. Determination of the Phagocytic Rate of Bovine Alveolar Macrophages

To determine the effects of recombinant proteins E2, E2Ft, and E2Fc on the phagocytosis of bovine alveolar macrophages (BAM), bovine alveolar macrophages at a concentration of 1 × 10^6^ cells/mL were pre-incubated with recombinant proteins E2, E2Ft, or E2Fc (the concentrations were all adjusted to 350 ng/mL) for 4 h. After washing three times with PBS, 10 μL of FITC-conjugated microspheres (Cat NO. 17155, Polysciences, Warrington, PA, USA) and 990 μL of the RPMI-1640 medium were added to each well. After 2 h of incubation, cells were washed with PBS to remove extracellular FITC-conjugated microspheres. Adherent macrophages were digested with trypsin (Gibco) containing 0.05% EDTA, and the digestion was terminated with the 1640 complete medium after full digestion. Cells were then added to the top layer of 10 mL PBS with 0.45 g glucose and 0.3 g BSA, followed by gradient centrifugation at 400× *g* for 10 min. Separated cells were washed twice with PBS and resuspended with 800 μL PBS. Fluorescence intensity at 488 nm was determined by the Beckman flow cytometer ( MoFloAstrios EQ, BECKMAN, Pasadena, CA, USA).

### 4.9. Vaccine Preparation and Immunization Procedure

HEK293-expressed E2, E2Ft, and E2Fc were emulsified with IMS 1313VG adjuvant (Seppic, France) in equal proportions to a final protein concentration of 200 μg/mL, and stored at 4 °C after the quality inspection. BVDV-E0 and BVDV-E2 antibody-negative (Cat NO. SU-AN78401, Win-win Biotechnology Co., Ltd., Shanghai, China) 3-month-old calves (n = 35) were divided into seven equal groups. Calves in four groups were nasal-mucosal immunized (i.n.) with PBS, E2, E2Ft, and E2Fc, respectively. Calves in the remaining three groups were intramuscularly injected (i.m.) with E2Ft, E2Fc, and BVDV inactivated vaccines as the positive control (Cat NO. 202002003, Huaweit, Jiangsu, China). Calves were vaccinated on days 1, 21, and 35 of the experiment, and each calf was inoculated with 1 mL of vaccine with a protein concentration of 200 μg/mL. Blood was collected from calves on days 1, 21, 35, and 49 of the experiment for other indexes (Figure 3A). All experimental protocols were approved by the Research Ethics Committee of the College of Veterinary Medicine, Huazhong Agricultural University, Hubei, China (No. HZAUCA-2022-0011).

### 4.10. Production of IgA in the Feces and Serum

Nasal swabs, feces, and serum samples of calves were collected on day 14 after the secondary immunization. The fresh cow feces were diluted into a 15% fecal suspension with PBS, the suspension was centrifuged at 18,000× *g* at 4 °C for 30 min, and the supernatant was collected for later use. The nasal swabs were collected by rotating the sterile cotton swab deep into the nasal cavity of the cow for 6–8 turns. Swabs were put into a PBS tube containing 1% penicillin–streptomycin and mixed extensively. Samples were centrifuged at 18,000× *g* at 4 °C for 10 min, and the supernatant was collected for later use. Bovine blood samples were collected with an ethylenediaminetetraacetic acid (EDTA)-containing anticoagulant tube, and the serum samples were obtained by centrifuging the blood samples at 4 °C and 400 g for 5 min. IgA (Cat NO. 177090b, CAMILO, Nanjing, China) and SIgA were detected with the ELISA kit (Cat NO.177053b CAMILO, Nanjing, China) according to the manufacturer’s instructions.

### 4.11. Flow Cytometry Analysis

The frequencies of IFN-γ-producing CD3^+^ CD4^+^ and CD3^+^ CD8^+^ T cells among the CD3^+^ lymphocytes in the blood were analyzed using flow cytometry. Samples were collected 14 days after the booster immunization [36]. Peripheral blood mononuclear cells (PBMC) were resuspended and diluted to 1 × 10^6^ cells/mL with complete RPMI-1640 medium, stimulated with UV-inactivated BVDV XZ02 (MOI = 0.1), and incubated at 37 °C with 5% CO_2_ for 72 h. Concanavalin A (Con A) (5 μg/mL; Sigma) was used as a positive control. The protein transport inhibitor brefeldin A (BFA, 3 μg/mL, eBioscience) was added to all samples 12 h before cell harvesting to block IFN-γ secretion. An amount of 200 μL of cell was transferred to FCM tubes and stained with the FITC-labeled CD4 antibody (Cat NO. MCA1653, BIO-RAD, Hercules, CA, USA) for 30 min at 4 °C in the dark. After being washed three times with PBS, cells were incubated with R-PE-labeled CD8 antibody (Cat NO. MCA837, BIO-RAD, Hercules, CA, USA). After being washed three times, cells were incubated with the mouse anti-bovine IFN-γ antibody (Cat NO. MCA1783A, BIO-RAD, Hercules, CA, USA) for 20 min in the dark for intracellular cytokine staining. After washing, cells were resuspended in PBS containing 1% FBS. Flow cytometry was conducted with the FACS Caliber flow cytometer (BD Biosciences, Franklin Lakes, NJ, USA), and data were collected and analyzed as previously described [38].

### 4.12. Lymphocyte Proliferation and Cytokine Detection

The anticoagulated peripheral blood mononuclear cells (PBMC) of each calf were isolated using a lymphocyte separation medium (Cat NO. RNBJ9540, SIGMA, Saint Louis, MO, USA) on day 49 after immunization. Bovine peripheral blood lymphocytes were diluted with the 10% FBS 1640 medium to 1 × 10^6^ cells/mL. Then, 100 µL of diluted cells was added to each well of a 96-well plate and stimulated with UV-inactivated BVDV, concanavalin (positive control), or culture medium (negative control), respectively. After 72 h of incubation at 37 °C with 5% CO_2_, 30 μL of MTS was added to each well, and cells continued to be cultured at 37 °C for 1 h according to the manufacturer’s instructions. The absorbance at 492 nm was measured with an automated microplate analyzer. The lymphocyte proliferation titer of each sample was determined by stimulation index (SI). Calculated SI = (OD of sample-OD of blank control)/(OD of negative sample-OD of blank control) [39]. Interferon (IFN)-γ, interleukin (IL)-2, and interleukin (IL)-4 were detected using ELISA kits. Briefly, PBMCs were isolated and diluted to 4 × 10^6^ cells/mL with RPMI 1640 medium containing 10% FBS and placed in 24-well tissue culture plates. Cells were stimulated with inactivated BVDV XZ02 (MOI = 1) for 48 h at 37 °C, and the supernatant was subsequently collected. The IFN-γ (Cat NO. 177020b, CAMILO, Nanjing, China), IL-2 (Cat NO. 177028b, CAMILO, Nanjing, China), and IL-4 (Cat NO. 1770131b, CAMILO, Nanjing, China) levels were detected using corresponding ELISA kits according to the manufacturer’s instructions The content of IFN-γ, IL-2, and IL-4 in the sample was calculated using the standard curve.

### 4.13. Serological Tests

To detect E2/E0 antibodies, blood samples were collected from each calf on days 0, 21, 35, and 49 after immunization. Serum samples were collected by centrifuging blood samples at 1000× *g* for 20 min. Serum samples were processed according to the requirements of the BVDV E2 antibody detection kit (Cat NO. SU-AN78401, Win-win Biotechnology Co., Ltd., Shanghai, China). Microplates coated with the BVBV E2 antigen were equilibrated for 20 min at room temperature. Then, 50 μL of negative control, positive control, and serum samples were added to the microplate, respectively. An amount of 50 μL of horseradish peroxidase (HRP)-labeled detection antibody was subsequently added to the microplate and incubated at 37 °C for 60 min. After being washed five times with a washing buffer, a chromogenic solution was added to measure the absorbance of the microplate reader at 450 nm to determine the level of E2 antibody in each immunization group.

The fixed virus dilution serum method was employed to detect the neutralizing antibody level of serum samples. Briefly, the completely inactivated immune serum samples were diluted by 2^−1^ to 2^−12^, and 50 µL of serum at each dilution titer was added to 96-well plates. Subsequently, 50 µL of diluted virus solution containing the 200 TCID_50_-BVDV XZ02 virus was added to each well. Plates were gently shaken and mixed. After 1 h of incubation at 37 °C, MDBK cells with a concentration of 5 × 10^5^ cells/mL were added to 96-well plates and incubated at 37 °C in a humidified incubator with 5% CO_2_ for 72 h. To detect the neutralizing effect of serum samples, cells were treated with the E2-specific antibody as the primary antibody (Cat NO. orb312169, Biorbyt, Cambridge, UK) and FITC-conjugated goat anti-rabbit IgG as the secondary antibody, (Cat NO. AS011, ABclonal, Boston, MA, USA). A typical green fluorescent signal indicated the absence of neutralization. Photographs were taken under a fluorescence microscope (TE2000, Nikon, Tokyo, Japan). The serum neutralization titer was calculated according to the Reed–Muench method, and the maximum dilution of serum that could completely inhibit fluorescence was determined as the neutralization titer of the serum.

### 4.14. Specificity Detection of BVDV E2 Monoclonal Antibody

Pre-absorption assays of BVDV E2 monoclonal antibody were first conducted, followed by viral replication assays and Western blot to testify the specificity of BVDV E2 mAb. For viral replication assay, 400 TCID_50_ of BVDV was pre-incubated with PBS and 100 μg or 200 μg of E2 mAb at 37 °C for 1 h, respectively. After incubation, the pre-treated BVDV was inoculated to MDBK cells, and cells were cultured at 37 °C in a humidified incubator. Cells were collected at 18 or 24 hpi (hour post infection) and RNA was extracted with TRIZOL reagent. RT-qPCR was conducted to determine the relative expression of the BVDV E2 gene. For Western blot assay, E2 mAb was pre-incubated with Tween20, PEDV S protein, or BVDV E2 protein in TBS-T for 2 h at 4 °C, respectively. Pre-treated E2 mAb was subjected to Western blot assay as the primary antibody to detect the reaction to the BVDV E2 or PEDV S protein.

### 4.15. Statistical Analysis

The statistical program GraphPad Prism was used for all analyses. The two-tail unpaired *t*-test was used to analyze the data and produce the *p* values (*, *p* < 0.05; **, *p* < 0.01; ***, *p* < 0.001, **** *p* < 0.0001). Except where otherwise noted, data are shown as mean ± SD.

## 5. Conclusions

In our study, three recombinant proteins, BVDV-E2, BVDV-E2Ft, and BVDV-E2Fc, were expressed, and corresponding subunit vaccines were generated. In vivo tests showed that the E2Fc subunit vaccine was more effective than E2Ft and E2, both in mucosal and intramuscular immunization. We confirmed that the neutralizing antibody titers induced by the mucosal-immunized E2Fc and E2Ft were 1:64 and 1:16, respectively, further indicating that E2Fc bonded with FcγRI on bovine alveolar macrophages to achieve transmucosal transportation. Mucosal vaccination of the multivalent E2Fc subunit vaccine of the main epidemic strain of BVDV significantly improved the immune effects, and it is suitable for practical use. Our study provides a solution to the time-consuming and labor-intensive conventional vaccination by employing mucosal vaccine. BVDV E2 protein is an ideal target for neutralizing antibodies, and the E2Fc and E2Ft fusion proteins constructed in this study are expected to be further applied as safe and effective subunit vaccines against BVDV infection.

## Data Availability

All data are contained within the article.

## Supplementary Materials

The following supporting information can be downloaded at: https://www.mdpi.com/article/10.3390/ijms24044172/s1.
