# Supplementary material for: Evaluation of the Mucosal Immunity Effect of Bovine Viral Diarrhea Virus Subunit Vaccine E2Fc and E2Ft
Source: Int J Mol Sci. 2023 Feb 20;24(4):4172. doi: 10.3390/ijms24044172 (PMC9965503; doi:10.3390/ijms24044172)
Supplement: Supplementary file 1 [file ijms-24-04172-s001.zip › Supplementary Figure S1.pdf]

Supplementary 1

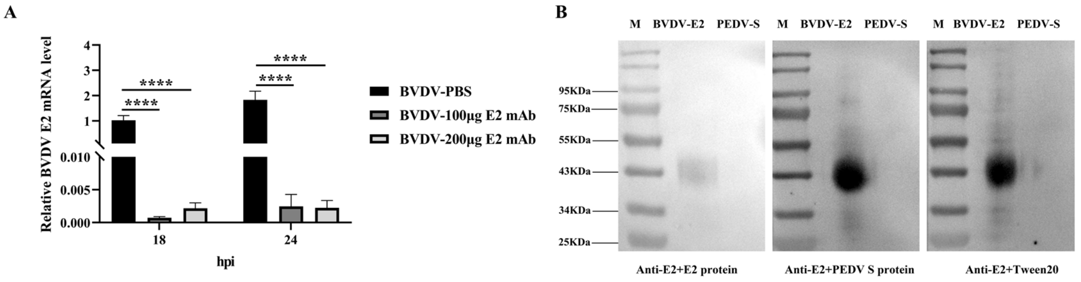

Figure S1. Specificity determination of BVDV-E2 mAb. (A) Pre-treated BVDV with PBS, 100μg or 200μg of E2 mAb, infected the MDBK cells and relative E2 gene expressions were detected by RT-qPCR at 18 and 24 hpi. (B) Western blot detection of the reaction to BVDV-E2 or PEDV-S protein using the pre-treated E2 mAb with BVDV E2 protein, PEDV S protein or Tween 20. BVDV: Bovine viral diarrhea virus, PEDV: Porcine epidemic diarrhea virus. hpi: hour post infection. *$p < 0.05$, **$p < 0.01$, ***$p < 0.001$ and **** $p < 0.0001$.
