# Supplementary material for: Evaluation of the Mucosal Immunity Effect of Bovine Viral Diarrhea Virus Subunit Vaccine E2Fc and E2Ft
Source: Int J Mol Sci. 2023 Feb 20;24(4):4172. doi: 10.3390/ijms24044172 (PMC9965503; doi:10.3390/ijms24044172)
Supplement: Supplementary file 1 [file ijms-24-04172-s001.zip › Supplementary Material S1.pdf]

Supplementary material S1: The nucleotide sequence of IL-2-E2-6XHis
ggatccgccaccatgtaccggatgcagctgctgagctgtatcgccctgagcctggccctggtgaccaacagcatgctgcc
cgcctgcaagcccgacttcagctacgccatcgccaagaacaacgagatcggccccctcggcgccaccggactgactaca
cagtggtacgaatatagcgacggcatgagactgcaagacaccgaggtggtcgtgtggtgcaaggatggcgagataaagt
acctgatcacatgcgaaagagaagccagatatctggcaatactgcacacccgggcactgcccacctccgtcgtgtttgaga
aaattataaagggcaaggagcaggaggacgtcgtggagatggacgacgacttcgagtttggactgtgcccctgcgacgc
caagccactcgttagaggcaagtttaacaccaccctgctgaacggccccgctttccagatggtgtgccccattggatggac
cggcaccgtgagctgcgcactcgccaacaaggacaccctcgccctgaccgtggtgagaacctacaccagacacaaacc
atttccctaccgccaaggctgtatcacccagaagactatcggagaagatctgtacaactgcgacctcggcggaaactggac
ctgcatccccggcgaccaactcagatacgtcgacggccccgtcgaaagctgcaagtggtgcggctataacttctacaaga
gcgagggcctgccccacttccctattggaaagtgcaaactgaagaacgagtctggctacagacaggtggacgagaccag
ttgcaacagagacggcgttgctatcgtgctgcacggcagggtgaaatgcaagatcggcgacaccgtggtgcaggtgata
gcaatggacgacagactcggacccatgccctgtataccccacgagatcattccttccgagggccccgttgagaaaacagc
ctgcaccttcaactataccaaaaccctgaagaataagtactacgagcccagagacaactacttccaacagtacatgctcaag
ggcgagtaccagtactggttcgacctggaagtgaccgaccaccacaaggactacttcgccgaaagcctgggcagcggca
gtcaccaccaccaccatcactgactcgag
