# Supplementary material for: Evaluation of the Mucosal Immunity Effect of Bovine Viral Diarrhea Virus Subunit Vaccine E2Fc and E2Ft
Source: Int J Mol Sci. 2023 Feb 20;24(4):4172. doi: 10.3390/ijms24044172 (PMC9965503; doi:10.3390/ijms24044172)
Supplement: Supplementary file 1 [file ijms-24-04172-s001.zip › Supplementary Material S3.pdf]

Supplementary material S3: The nucleotide sequence of IL-2-E2Ft-6XHis

ggatccgccaccatgtaccggatgcagctgctgagctgtatcgccctgagcctggccctggtgaccaacagcctgcccgc
ttgcaagcctgacttctcctacgctatcgctaagaacaacgaaatcggccccctcggtgctaccggtctgactacccagtgg
tacgagtactccgacggtatgcgcctgcaagataccgaggtggtcgtgtggtgcaaggacggcgaaatcaagtacctgat
cacctgcgaacgtgaagcccgctacctggccatcctgcacactcgcgctctgcctacctccgtcgtcttcgaaaagatcatc
aagggtaaggaacaggaggacgtcgtggagatggacgacgacttcgaatttggtttgtgcccttgtgacgctaagccactg
gtgcgcggtaagttcaacaccaccctgctgaacggccccgctttccagatggtgtgccctatcggatggaccggaaccgt
gagttgtgctctggctaacaaggacaccctggctttgactgtggtgcgcacctacacccgccacaagcctttcccctaccgc
caaggttgtatcacccagaagactatcggagaagatctgtacaactgcgacctcggcggtaactggacctgcatccctggt
gaccaactccgctacgtcgatggtcctgtcgagtcctgcaagtggtgcggctacaacttctacaagagcgagggactgcct
cacttccctatcggtaagtgtaagctgaagaacgagagtggttaccgccaggtggacgagaccagctgcaaccgcgacg
gtgtggctatcgtgctgcacggacgcgtgaagtgcaagatcggtgacaccgtggtgcaggtgatcgccatggacgacag
gctgggtcctatgccttgcatcccccacgagatcatcccttctgagggtcctgtggagaagactgcttgcaccttcaactaca
ccaagaccctgaagaacaagtactacgagccacgcgacaactacttccagcagtacatgctgaagggcgagtaccagta
ctggttcgatctggaagtgaccgaccaccacaaggactacttcgccgaatccctcggaggcggctctatgctctccaagga
catcatcaagttgctgaacgagcaggtgaacaaggagatgcagtcctccaacctgtacatgtctatgtcctcctggtgctaca
cccacagcctggacggtgctggcctcttcctgttcgaccacgccgctgaagaatacgaacacgccaagaagttgatcatct
tcctgaacgagaacaacgtgcctgtccagctcaccagcatctccgctcctgagcacaagttcgagggtctgacccaaatctt
ccagaaggcttacgaacacgagcagcacatctccgagtccatcaacaacatcgtggaccacgccatcaagtccaaggac
cacgctaccttcaacttcttgcagtggtacgtcgccgagcaacacgaagaagaagtcctgttcaaggacatcctggacaag
atcgagctgatcggaaacgagaaccacggtctgtacctcgctgaccagtacgtgaagggtatcgccaagtcccgcaagtc
cggcagcggcagtcaccaccaccaccatcactgactcgag
